# Comprehensive watermelon disease recognition dataset

**DOI:** 10.1016/j.dib.2024.110182

**Published:** 2024-02-13

**Authors:** Mohammad Imtiaz Nakib, M.F. Mridha

**Affiliations:** Department of Computer Science, American International University-Bangladesh, Dhaka, Bangladesh

**Keywords:** Image recognition, Agriculture, Watermelon dataset, Deep learning, Computer vision

## Abstract

Plant diseases pose a significant obstacle to global agricultural productivity, impacting crop quality yield and causing substantial economic losses for farmers. Watermelon, a commonly cultivated succulent vine plant, is rich in hydration and essential nutrients. However, it is susceptible to various diseases due to unfavorable environmental conditions and external factors, leading to compromised quality and substantial financial setbacks. Swift identification and management of crop diseases are imperative to minimize losses, enhance yield, reduce costs, and bolster agricultural output. Conventional disease diagnosis methods are often labor-intensive, time-consuming, ineffective, and prone to subjectivity. As a result, there is a critical need to advance research into machine-based models for disease detection in watermelons. This paper presents a large dataset of watermelons that can be used to train a machine vision-based illness detection model. Images of healthy and diseased watermelons from the Mosaic Virus, Anthracnose, and Downy Mildew Disease are included in the dataset's five separate classifications. Images were painstakingly collected on June 25, 2023, in close cooperation with agricultural experts from the highly regarded Regional Horticulture Research Station in Lebukhali, Patuakhali.

Specification Table

**Table:** 

Subject	Computer Science
Specific subject area	Plant Disease Recognition Using Computer Vision and Deep Learning
Type of Data:	Image
Data collection	We captured images of both healthy and diseased watermelon plants in real field conditions between June 15 and June 25, 2023, using an iPhone 13 Pro Max to acquire the original image data.
Data source location	Institution: Regional Horticulture Research Station, Lebukhali, Patuakhali. Zone: Patuakhali, Barishal
Data accessibility	Data identification number (DOI number): 10.17632/ntzym554jp.1 Link of the dataset: https://data.mendeley.com/datasets/ntzym554jp/1

## 1 Value of data

• Farmers and distributors lose time and money due to the inefficiency of manually identifying watermelon diseases. Hence, the imperative in the agriculture industry is to minimize human labor, cut down expenses, and accelerate production. This makes the development of a machine vision-based model for consistent recognition of multiple watermelon diseases essential.. Building a better classification model requires a substantial dataset.
• To facilitate the development of efficient agricultural automation systems, This dataset showcases the visual appearances of watermelon diseases through a sequence of hyperspectral images.
• The images captured can be used to train, test, evaluate, and distinguish various deep learning models for disease detection by utilizing multiple features.
• This collection's original watermelon disease photographs offer a broader viewpoint than the binary classes (healthy and sick) and sparse data of prior datasets [1,2]. This comprehensive approach makes it easier to identify numerous watermelon illnesses, improving performance and assisting plant physiologists in building automated disease recognition systems.
• The photographs were taken in authentic fields under natural weather circumstances with varying lighting, making it difficult for researchers to diagnose ailments with the human eye properly.

## 2 Background

The main aim of this article is to present a comprehensive watermelon dataset that encompasses a wider spectrum of disease categories [1]. This dataset will be used to build a powerful machine vision-based recognition algorithm capable of diagnosing various watermelon diseases independently. This initiative aims to improve agricultural efficiency, raise output levels, and ensure food security.

## 3 Dataset Description

Watermelons, members of the Cucurbitaceae family, are celebrated for their refreshing flavor and their ability to help combat dehydration, particularly in hot summer months, as they are comprised of approximately 92% water. Nevertheless, the cultivation of watermelons faces a multitude of challenges, notably the potential threat of various diseases that can significantly harm crop yields. Regrettably, these diseases often remain undetected by farmers without the aid of specialized equipment, leading to substantial economic losses. Additionally, farmers' limited access to training and education further impedes their capacity to seek guidance from agricultural experts when confronted with such issues [2].

Watermelons play an integral role in the agricultural sector, serving not only as a beloved summer treat but also as a crucial contributor to global food security. Given the persistent challenges associated with watermelon cultivation, the dataset mentioned earlier emerges as a valuable tool for the development of advanced machine vision algorithms. These algorithms are designed to identify and categorize watermelon diseases at an early stage, ultimately safeguarding the sustainability of watermelon production and its pivotal role in agriculture.

We provide a comprehensive watermelon dataset for developing machine vision-based algorithms, including four distinct watermelon classes (Mosaic Virus, Healthy, Anthracnose, and Downy Mildew). There are 1155 unique photos in this watermelon dataset. The representative images for the four classes are listed in Table 1. Find which diseases are of interest for inclusion in the dataset. This could involve utilizing current statistics as a jumping-off point, talking with professionals in the sector, or undertaking research to uncover frequent watermelon diseases. Once the specific illnesses of interest have been pinpointed, we gather samples of plants afflicted with those diseases.

**Table 1:** Statistical information regarding the watermelon dataset.

Category	Number of original images	Number of augmented images
Mosaic Virus	415	2075
Downy Mildew	380	1900
Anthracnose	155	775
Healthy	205	1025
**Total**	**1155**	**5775**

In our image dataset augmentation process, we employ a set of diverse techniques to enrich the dataset for improved object recognition in computer vision applications. These techniques include horizontal flipping, both left and right, which mirrors images along the vertical axis, thereby expanding the dataset's orientation diversity. Scaling plays a crucial role, with images randomly adjusted in the *X*-axis (horizontal) within a range of 1.0–2.5 times their original width and in the *Y*-axis (vertical) within a range of 0.7–1.0 times their original height. This scaling introduces variations in object size and aspect ratio. Additionally, we apply brightness adjustments by randomly altering image brightness within the range of 0.8–1.8, simulating diverse lighting conditions. Finally, zooming is incorporated, with a zoom factor chosen randomly between 1.0 and 1.5, enabling the dataset to encompass different object scales. These augmentation techniques collectively create a more versatile and robust dataset, enhancing object recognition models’ ability to adapt to real-world variations and challenging scenarios. The watermelon dataset is available for download on the Mendeley repository. It is structured into two primary folders: one for the original images and another for the augmented images. Inside each of these folders, you'll find four subfolders, each representing one of the four distinct data categories: Mosaic Virus, Healthy, Anthracnose, and Downy Mildew. To simplify the download process, we have compressed the contents of these two folders into zip files named 'Augmented Image.zip’ and 'Original Image.zip.

## 4 Experimental Design, Materials & Methods

### 4.1 Camera specification

**Fig. 1:**
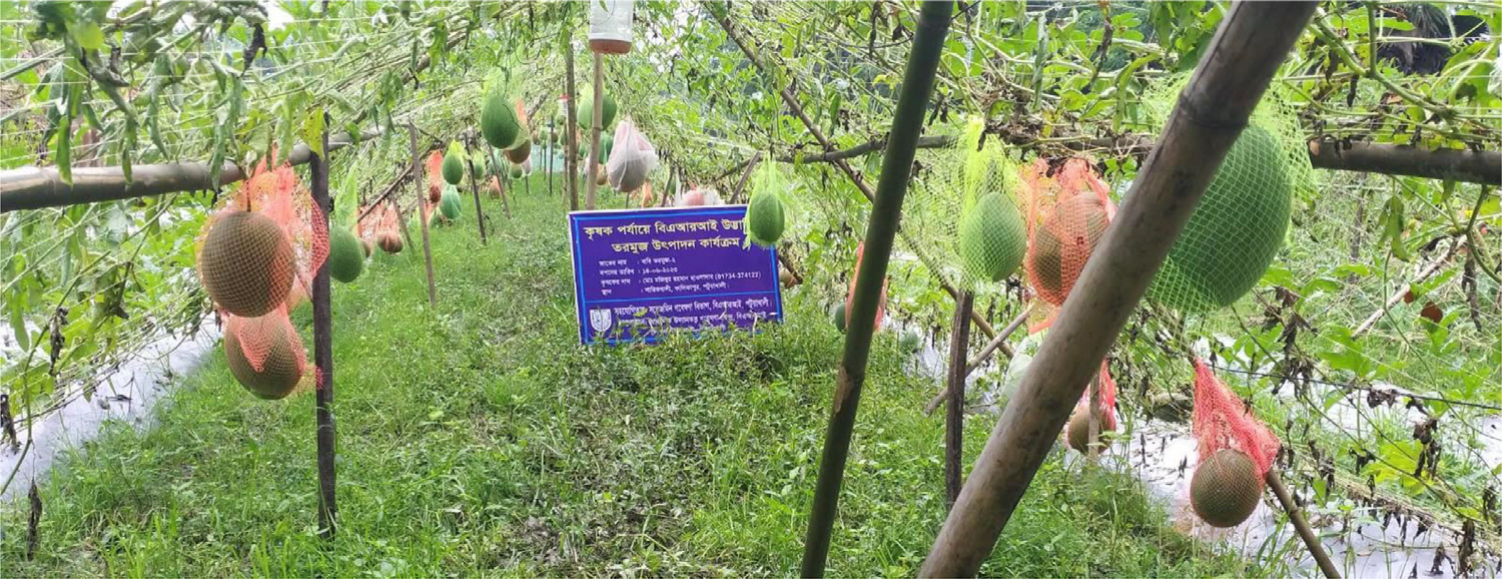
The data were collected from a real watermelon field.

**Fig. 2:**
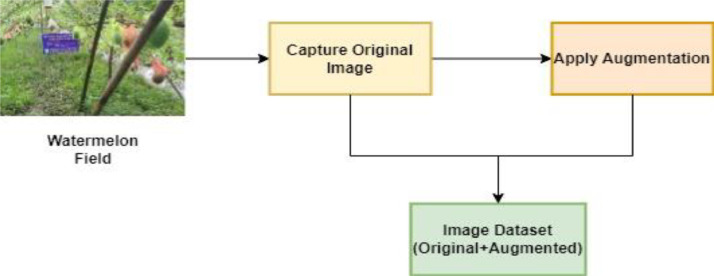
Watermelon disease dataset generation.

I phone 13 pro max Primary camera which is 12 MP sensor, 1.9µm pixels, 26 mm equivalent f/1.5-aperture lens, sensor-shift OIS, Dual Pixel AF is used to capture all the images (Figs. 1, and 2).

### 4.2 Deep learning model validation

**Fig. 3:**
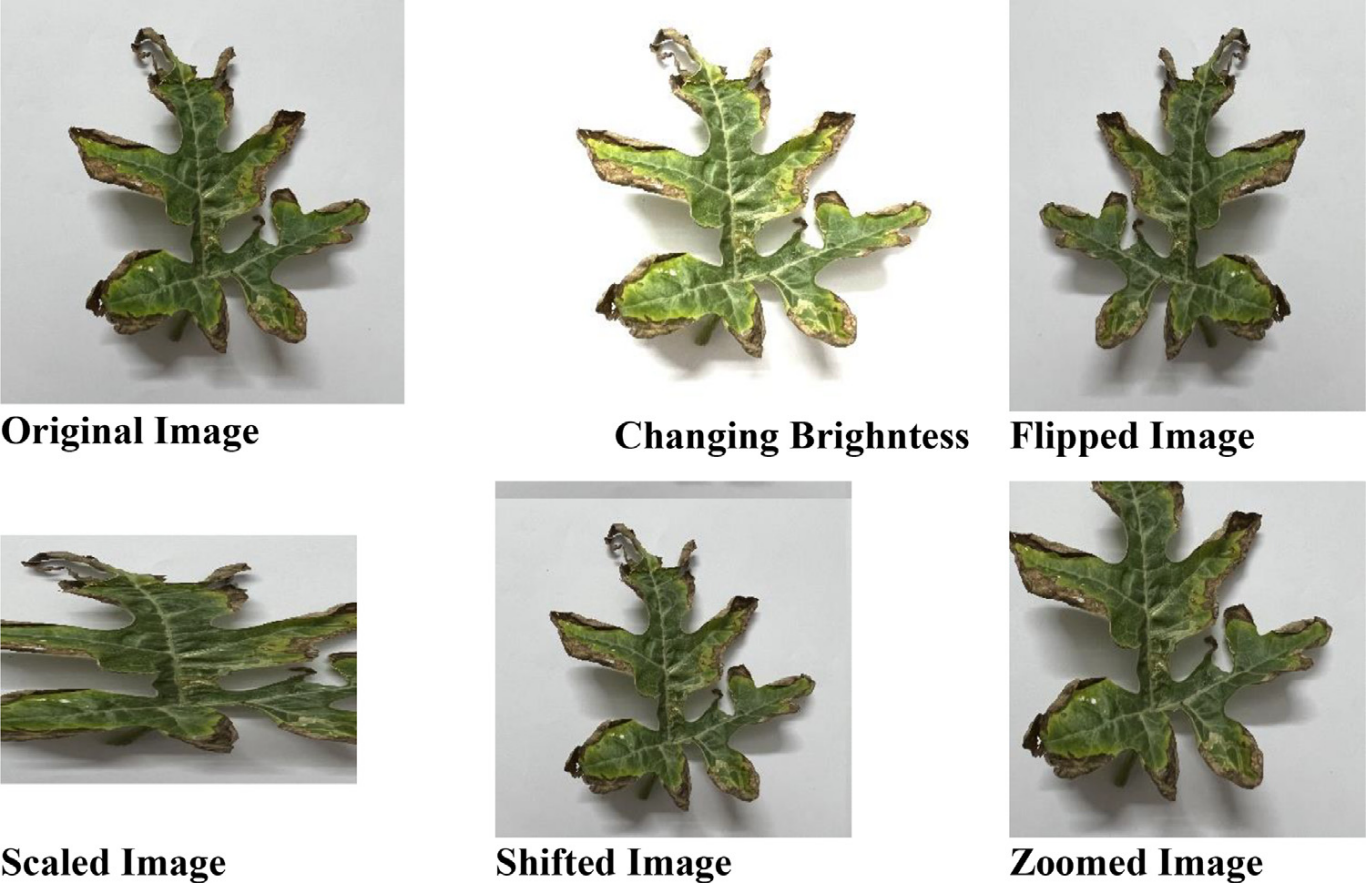
Augmented images.

**Fig. 4:**
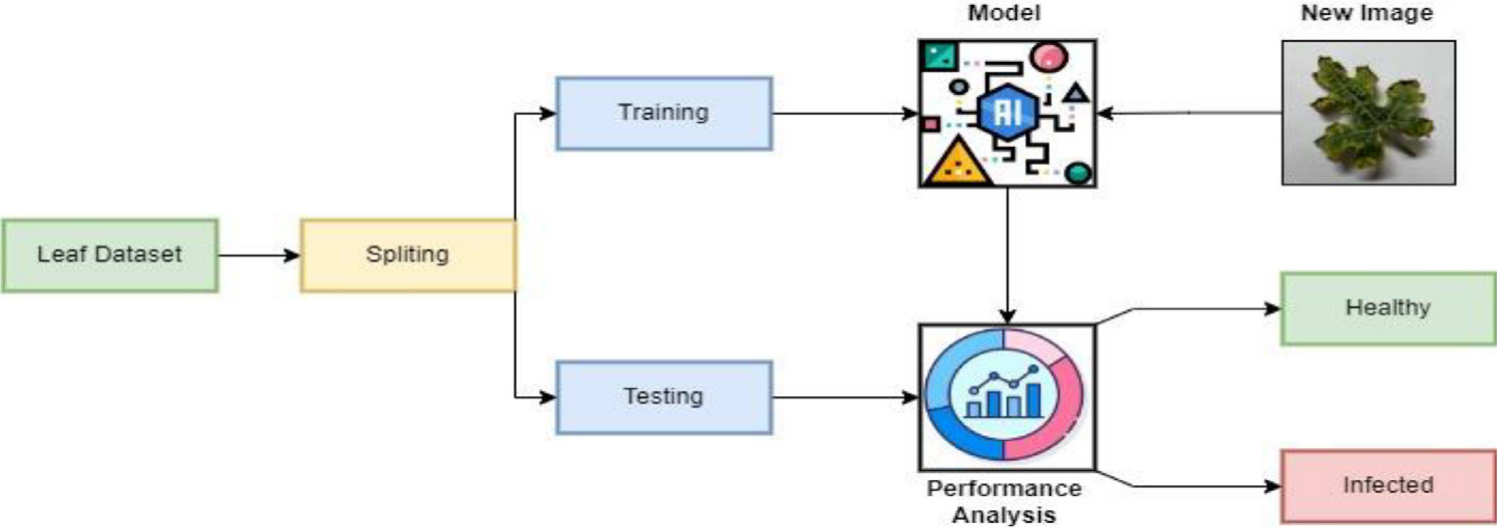
Generic working process for recognition of watermelon diseases.

We use tried-and-true deep-learning training methods on a large-scale dataset to get state-of-the-art results. Data must be prepared, partitioned, a model trained, and its performance evaluated on a validation subset. Then, it must be tested on a separate test subset before validation can be considered complete. This rigorous approach ensures that the model can generalize and provide accurate outcomes. Data preprocessing includes scaling, normalization, and augmentation [3]. The dataset is divided into training and test sets, and we focus on training the watermelon dataset using CNN and Densenet121 models, achieving impressive accuracies of 96% and 98%, respectively. This confirms the models' proficiency in unseen data, as depicted in Fig. 2 (Figs. 3 and 4) (Table 2).

**Table 2:** Description of four classes of sample images in the watermelon dataset.

Class name	Description	Visualization
Mosaic Virus	The watermelon mosaic virus is a potyvirus that causes a variety of symptoms, including leaf chlorosis with a yellow or green mosaic pattern, rapid growth, and deformed leaves. The leaves begin to stunt, have yellow spotting, and are also smaller. The leaves twist as a result of the plant's stunted growth, and fruit yield is typically minimal.	
Downy Mildew	Downy mildew on watermelon plants is caused by Pseudoperonospora cubensis, which shows symptoms like leaf yellowing, angular lesions, and greyish downy development beneath leaves. It spreads via tools, water, wind, and humidity at 10–24 °C (50–75°F), where it thrives. For effective control, management entails picking resistant varieties, spacing, and reducing overhead watering, along with fungicides and routine inspections. Local experts offer specialised treatment suggestions.	
Anthracnose	Anthracnose, caused by Colletotrichum orbiculare, affects watermelon plants, causing lesions on leaves, vines, and fruit. It spreads via water splashes, wind, and contaminated equipment, with optimal growth between 72°F and 80°F (22 °C and 27 °C). It can survive in plant debris and seeds. Managing it involves using disease-free seeds, crop rotation, clean cultivation, and appropriate fungicides.	
Healthy Leaf	A bright green colour, a smooth texture, turgidity, and the absence of extensive yellowing or brown necrotic patches are all signs of a healthy watermelon leaf. It should have healthy leaves, be free of damage or bugs that can be seen, and grow consistently. The entire health and productivity of the plant can be ensured through routine examination and rapid response**.**	

## Limitations

We faced a number of obstacles in our effort to create an extensive dataset for the identification of watermelon illness. Unfavorable weather conditions made fieldwork difficult, and finding appropriate farms was first difficult. Iterative refinement was necessary because to technical issues that developed throughout the dataset augmentation process. Despite these difficulties, we were able to overcome them thanks to our cooperation with agricultural specialists and strict quality control procedures. We've developed a useful tool to enhance machine vision algorithms in agricultural contexts via persistence and optimisation work. These experiences highlight the difficulty of such undertakings and stress the value of perseverance and teamwork in overcoming challenges [4].

## CRediT authorship contribution statement

**Mohammad Imtiaz Nakib:** Conceptualization, Methodology, Data curation. **M.F. Mridha:** Visualization, Supervision.

## Data Availability

WM-DATASET: A Comprehensive Dataset for Watermelon Disease Recognition (Original data) (Mendeley Data).
